# Evaluating the clinical care, quality of life and overall experiences of patients with primary biliary cholangitis (PBC) during the pandemic: A Canadian mixed-methods study

**DOI:** 10.1371/journal.pone.0340475

**Published:** 2026-01-09

**Authors:** Elizabeth Baguley, Madelyn Knaub, Jessica VanDyke, Gideon Hirschfield, Mark G. Swain, Gail Wright, Deirdre McCaughey, Abdel Aziz Shaheen

**Affiliations:** 1 University of Calgary, Division of Gastroenterology and Hepatology, Calgary, Alberta, Canada; 2 University of Calgary, Department of Community Health Sciences, Calgary, Alberta, Canada; 3 University of Calgary, Ward of the 21st Century (W21C), Calgary, Alberta, Canada; 4 Toronto Centre for Liver Disease, Division of Gastroenterology and Hepatology, University of Toronto, Toronto, Ontario, Canada; 5 Canadian PBC Society, Toronto, Ontario, Canada; Laval University, CANADA

## Abstract

Pandemic restrictions impacted healthcare, particularly during the first year. We evaluated the impact of the pandemic on quality of life and clinical care among patients with primary biliary cholangitis (PBC). This mixed-methods study administered quality of life surveys (Fear of COVID-19 Scale [FCV-19S], EuroQol 5-dimension 3-level [EQ-5D-3L], 29-item Patient-Reported Outcomes Measurement Instrument Survey [PROMIS-29]) and a PBC Care Delivery questionnaire to 348 Canadian PBC patients, followed by two focus groups with patients (n = 14) and stakeholders (n = 3). Quality of life scores were compared among sub-groups (i.e., care delays and pandemic appointment type) and with various reference populations. Most participants were female (94.0%) and Caucasian (88.2%), with a median age of 63.0 years (IQR: 55.9–71.2). During the pandemic, 75.8% had the majority (≥ 50%) of their hepatologist appointments virtually, but only 22.4% preferred to continue with virtual care post-pandemic. Participants with care delays had worse scores on the FCV-19S (*p* = 0.014), EQ-5D-3L (*p* = 0.009), and PROMIS-29 (i.e., fatigue, anxiety, sleep disturbance, ability to participate in social roles and activities, *p* < 0.01), compared to those without care delays. PBC patients had worse PROMIS-29 scores compared to a general population (*p* < 0.01). Both patients and stakeholders stressed the importance of in-person appointments, while recognizing a role for virtual appointments post-pandemic. PBC patients’ quality of life worsened during the pandemic, especially those with delayed care. Most PBC patients expressed a preference for in-person appointments post-pandemic.

## Introduction

Primary Biliary Cholangitis (PBC) is a chronic autoimmune liver disease that causes inflammation and fibrosis of intrahepatic bile ducts, potentially leading to end-stage liver disease [1]. PBC affects women at a rate nine times higher than men, and while approximately 60% of patients are asymptomatic at diagnosis, only 5% remain asymptomatic over their lifetime [2,3]. Symptoms associated with PBC, including pruritus, fatigue, and cognitive impairment, can significantly impact quality of life [4–6]. Although treatments for PBC, such as fibrates, may reduce symptoms (i.e., itch and fatigue), about 30% of patients may not respond or tolerate these medications [7,8]. The management of PBC often involves frequent healthcare visits, including appointments with a PBC care provider (i.e., hepatologist), symptom management, medication access, and ongoing investigations (i.e., lab work and imaging) [6,9].

The coronavirus disease 2019 (COVID-19) pandemic disrupted medical care for all patients [10]. Healthcare facilities, including laboratories and imaging departments, limited public access, and patients were hesitant to seek care due to the risk of contracting the virus, ultimately leading to care delays [11]. As a result, many healthcare providers transitioned to virtual care, offering video and telephone appointments [12,13].

Previous literature evaluating the quality of life among PBC patients during the pandemic is limited. Further, virtual care models have shown both benefits and challenges in other medical conditions, depending on factors such as work commitments or geographical location, but remains underexplored in PBC [14–16]. This study aims to assess the quality of life and hepatology care provision among patients with PBC during the first year of the pandemic, and understand how virtual care models might best contribute to post-pandemic healthcare.

## Methods

### Study population and setting

**Fig 1:**
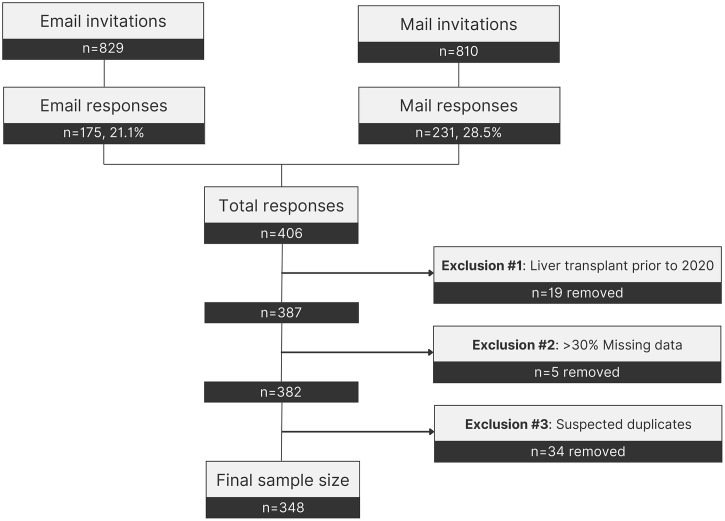
Patient recruitment and inclusion flowchart.

This cross-sectional mixed-methods study included 348 Canadian PBC patients and 3 stakeholders from August 2021 to August 2022. We partnered with the Canadian PBC Society, a national registered charity, for participant recruitment. An invitation to complete an online survey was sent to 829 valid email addresses on the Society’s email list on August 24^th^, 2021, with 175 participants responding. A follow-up advertisement with the survey link was posted on the Society’s Facebook page on September 15^th^, 2021. On November 12^th^, 2021, paper copies of the survey were mailed with return labels to 810 Society members, resulting in 231 responses. The last survey was received on August 17^th^, 2022, marking the end of the recruitment period. A total of 406 responses were received from all modes. After excluding patients with a liver transplant prior to pandemic restrictions (March 2020) (n = 19), responses with over 30% missing data (n = 5), and suspected duplicate responses (n = 34), 348 surveys were eligible for our study and were analyzed (Fig 1). Survey participants interested in the focus groups submitted their emails, and participation was offered in sequential order by a researcher (EB). A consent form for the survey portion of the study was presented to each patient prior to completing the survey. The survey consent form was either an online copy that participants had to review before continuing to complete the survey, or a paper copy that was mailed with the survey contents to participants mailing addresses. Consent to participate in the survey was implied if the patient completed the survey online or returned the paper survey responses. A focus group consent form was developed and shared with the participants who expressed interest in participating in the focus group discussions. At the beginning of each focus group session, the consent form was reviewed and participants were given the opportunity to ask questions. Verbal informed consent was obtained prior to initiating audio recording and documented by the study team. Participants could withdraw at any point during the discussion. No minors participated. This study was approved by the Conjoint Health Research Ethics Board at the University of Calgary (REB20–2155).

### Quantitative methods & data analysis

The online survey assessed patient healthcare experiences with their PBC provider (herein, referred to as hepatologist), preferences for care provision (i.e., in-person versus virtual), and evaluated quality of life during the first year of the pandemic (i.e., March 2020 to March 2021). The PBC Care Delivery questionnaire was developed by two hepatologists (AAS and MS) and a Canadian PBC Society representative (GW), as to date, no survey has been developed and validated to assess PBC patients’ views of the care provided to them. Additionally, three validated quality of life questionnaires were used: Fear of COVID-19 scale (FCV-19S), EuroQol 5-dimension 3-level (EQ-5D-3L), and the 29-item Patient-Reported Outcomes and Measurement Information System (PROMIS) Profile (PROMIS-29) (Version 2.0) [17–19].

We selected the EQ-5D-3L, PROMIS-29, and FCV-19S to align with our aim of evaluating pandemic-related impacts on quality of life and to enable comparisons with general population reference values. Although PBC-40 is validated in patients with PBC, it does not capture several domains relevant to the pandemic context (e.g., physical function, pain, sleep) and does not separate anxiety and depression. Generic instruments (EQ-5D-3L and PROMIS-29) provide established normative data, and FCV-19S quantifies fear of COVID-19 using a validated measure. Sociodemographic (i.e., age, gender, ethnicity) and PBC-related care variables were collected by the PBC Care Delivery questionnaire (S1 File). Participants were asked about their PBC diagnosis and staging (i.e., presence of cirrhosis), along with any known decompensated cirrhosis-related conditions (i.e., ascites, variceal bleeding, jaundice, and hepatic encephalopathy). Respondents were asked during the first year of the pandemic (i.e., March 2020 to March 2021) whether they received virtual or in-person care, and whether they experienced delays in medical, bloodwork, procedural appointments (i.e., Transient Elastography [Fibroscan^®^], abdominal ultrasound, bone density scans) or access to medications. Lastly, patients were prompted to indicate their future preference between virtual and in-person appointments for post-pandemic PBC-related care.

A respondent’s fear of COVID-19 was assessed using the FCV-19S. The FCV-19S is a 5-item Likert type scale, where respondents indicate their level of agreement with statements surrounding COVID-19, on a scale of 1–5, “strongly disagree” to “strongly agree”, respectively [17]. A total score is determined by summing all scores of each statement, ranging from 7 to 35, where a higher score is indicative of greater fear toward COVID-19 [17]. For participants who did not fill out all questions, a summed score was not calculated and their values were treated as missing. Previous literature has used FCV-19S to assess fear of COVID-19 infection and its relationship with quality of life, where findings suggest that higher FCV-19S scores are negatively associated with quality of life [20]. General reference population median and interquartile range (IQR) data for FCV-19S was not available to compare to our cohort.

The EQ-5D-3L survey assesses quality of life measures in five domains, including mobility, self-care, usual activities, pain and discomfort, and anxiety and depression, followed by an overall health scale (visual analog scale [VAS]) ranging from 0 (the worst health you can imagine) to 100 (the best health you can imagine). Respondents report the most appropriate level for each domain out of three possible levels: no problems, some problems, and extreme problems [18]. Each participant’s answers were combined into a health state based off the level they chose for each of the five questions in the survey (i.e., 11111, 12111, 12311, etc.) [21]. The corresponding value associated with the health state was assigned to each participant from the Canadian Valuation [21]. Individuals that did not fully complete the EQ-5D-3L were treated as missing. EQ-5D median scores were compared to pre-pandemic PBC reference population data [22].

The PROMIS-29 survey covers a range of health domains, including physical function, fatigue, pain interference, sleep disturbance, depression, anxiety, and ability to participate in social roles and activities. Each question is measured from 1 to 5, and a single pain intensity item that is measured from 0 (no pain) to 10 (worst imaginable pain) [19]. The PROMIS domains of physical function and ability to participate in social roles and activities are positively worded, meaning a higher score is better on these two instruments. The PROMIS domains of anxiety, depression, fatigue, sleep disturbance, and pain interference are negatively worded, meaning a higher score is worse for these instruments. Every PROMIS domain, besides the numeric pain intensity scale, receives a T-score (i.e., ranging from 0–100). The PROMIS-29 T-scores were computed using an online scoring system available at www.healthmeasures.net [19]. The scoring service can calculate T-scores with missing responses, as long as one question per domain was completed. To obtain the T-scores from the scoring service, one researcher (EB) submitted an anonymized excel sheet with the raw answers for each participant’s PROMIS domains. Finally, the PROMIS Pain Intensity Numeric Rating Scale [0–10] was treated as raw data and not given a T-score. Some participants did not answer this question and therefore answers were treated as missing values. PROMIS-29 data were compared to a general reference population collected from the 2000 United States (USA) census [19]. General reference population data is purposefully centered at mean/median T-score of 50. Since general population data on the PROMIS numeric pain intensity scale is not available, we did not compare this metric against a reference population.

Data are presented as median (IQR) or mean with standard deviation (SD) depending on skewness for continuous data, or percentage (count) for categorical data. Data comparisons were considered statistically significant with an alpha value <0.05. Statistical analyses included Wilcoxon rank-sum test, two-sample t-test, chi-square test and Wilcoxon sign-rank test. Univariable and multivariable logistic regression were used to identify demographic and clinical characteristics associated with a preference for virtual care post-pandemic. Exposure variables with a *p*-value <0.05 from the univariable logistic regression were used in the multivariable logistic regression. Demographic and clinical variables in the univariable and multivariable regression included age, length of time with PBC diagnosis, diagnosis of liver cirrhosis, decompensated liver disease-related conditions, number of times seen by hepatologist during the pandemic, experienced any delays in PBC-related care during the pandemic, location of hepatologist, distance to hepatologist office, pandemic appointment type, FCV-19S, EQ-5D-3L, EQ-5D VAS and all PROMIS domains. Age was included in the multivariable logistic regression models, regardless of significance in the univariable model. All survey data were analyzed using STATA Version 18.0.

### Qualitative methods & data analysis

Two focus groups of up to nine patients each were conducted in June and August 2022 (n = 14) to allow for comprehensive and exhaustive discussion, with a separate group for stakeholders (n = 3) [23,24]. Discussion guides were informed by the quantitative data and developed collaboratively by two researchers (JVD) (EB). The stakeholder focus group included one representative from the Canadian PBC Society (GW) and two hepatologists (MS and GH). All focus groups were attended by two researchers (JVD and EB) and facilitated by JVD. Focus groups were held in English, conducted via Zoom online teleconferencing software and lasted between 60–90 minutes. At the beginning of each focus group, participants were introduced to the researchers, informed of the focus group purpose and given the opportunity to withdraw consent or ask any questions before initiating audio recording. Audio recordings were then de-identified and transcribed verbatim. NVivo 12 software was used for thematic analysis, where two researchers (JVD and EB) examined each focus group transcripts and agreed upon codes used to avoid any potential bias.

## Results

### Quantitative analysis

A total of 348 PBC patients provided consent and met inclusion criteria (Fig 1). The study sample was predominantly female (94.0%, n = 327) and Caucasian (88.2%, n = 307), with a median age of 63.0 years (IQR: 55.9–71.2) (Table 1). Participants had been diagnosed with PBC for a median of 9.2 years (IQR: 4.7–18.2). Before the pandemic, participants typically saw their hepatologist once per year (IQR: 1.0–2.0), which remained unchanged during the pandemic. Over a quarter participants had a diagnosis of cirrhosis (25.9%, n = 90). While many patients did not report features of decompensated cirrhosis (69.9%, n = 243), 8.9% (n = 31) reported having at least one condition, 6.3% (n = 22) reported having two or more conditions, and 10.6% (n = 37) were unsure. Five participants (1.4%) received a liver transplant during the pandemic.

**Table 1:** PBC patient characteristics (n = 348).

Continuous characteristics	Median (IQR^a^)
Age (years) (n = 301)	63.0 (55.9–71.2)
Time since PBC diagnosis (years) (n = 333)	9.2 (4.7–18.2)
Frequency that participant saw hepatologist *before* the pandemic (n = 336)	1 (1 –2 )
Frequency that participant saw hepatologist *during* the pandemic (n = 330)	1 (1 –2 )
**Categorical characteristics**	**% (n)**
Gender	
Female	94.0% (327)
Male	4.9% (17)
Gender Variant/Non-Conforming	0.3% (1)
No response	0.8% (3)
Ethnicity	
Caucasian	88.2% (307)
Other	7.5% (26)
No response	4.3% (15)
Presence of liver cirrhosis	
Yes	25.9% (90)
No	54.0% (188)
Not sure	18.7% (65)
No response	1.4% (5)
Diagnosis of decompensated liver cirrhosis conditions^b^	
Never	69.9% (243)
Had one decompensated liver condition	8.9% (31)
Had more than one decompensated liver condition	6.3% (22)
Unsure	10.6% (37)
No response	4.3% (15)
Received liver transplant during study period	
Yes	1.4% (5)
No	98.0% (341)
No response	0.6% (2)

^a^Interquartile range.

^b^Decompensated liver conditions included ascites, variceal bleeding, jaundice and hepatic encephalopathy.

In the two patient focus groups, 14 female Caucasian participants with a median age of 63.0 years (IQR: 58.0–69.0) were included. The median duration of their PBC diagnosis was 9.2 years (IQR: 2.0–15.9), however two participants did not report date of diagnosis.

### PBC-related medical appointments before and during the pandemic

Before the pandemic, all hepatology appointments were in-person, with 60.3% (n = 210) attending tertiary care centers and 31.9% (n = 111) attending community clinics (Table 2). While the majority of participants travelled less than 10 km (36.0%, n = 117) or between 10–50 km (38.8%, n = 126) to their hepatology appointments, over a quarter travelled more than 50 km (25.2%, n = 82). During the first year of the pandemic, 75.8% had the majority (≥50%) of their hepatologist appointments through virtual and/or phone methods (n = 264). Only 11.5% (n = 40) had the majority (>50%) of their appointments in-person (Table 2). A total of 40 participants (11.5%) did not receive any hepatology care during the pandemic. For future hepatology appointments, 48.6% (n = 169) preferred not to return to virtual care, while 22.4% (n = 78) wanted to continue with virtual visits (Table 2). Delays occurred in routine lab work (22.4%, n = 78), imaging (23.6%, n = 82) and obtaining medication (5.7%, n = 20) (Table 2). Delays were distributed across the three domains, with 13.2% (n = 46) experiencing one, 12.9% (n = 45) experiencing two, and 1.7% (n = 6) experiencing all three delays (Table 2).

**Table 2:** Medical appointment characteristics of PBC patients (n = 348).

Medical Appointment Characteristic	% (n)
Hepatologist location	
Tertiary centre affiliated clinic (i.e., Hospital)	60.3% (210)
Community clinic	31.9% (111)
No response	7.8% (27)
Distance travelled to see hepatologist	
< 10km 0–50 km	36.0% (117) 38.8% (126)
> 50km	25.2% (82)
Hepatologist appointment type during the pandemic	
Majority (≥50%) virtual/phone	75.8% (264)
Majority (≥50%) in-person	11.5% (40)
Did not receive care	11.5% (40)
No response	1.2% (4)
Preference for future hepatologist virtual/phone appointments	
Yes	22.4% (78)
No	48.6% (169)
No preference	19.0% (66)
Not sure	9.5% (33)
No response	0.5% (2)
Experienced delays in routine lab work	
Yes	22.4% (78)
No	70.1% (244)
Not sure	1.4% (5)
No lab work requested	5.2% (18)
No response	0.9% (3)
Experienced delays in imaging investigations	
Yes	23.6% (82)
No	52.0% (181)
Not sure	2.6% (9)
No imaging requested	21.2% (74)
No response	0.6% (2)
Experienced delays in obtaining medications	
Yes	5.7% (20)
No	92.8% (323)
Not sure	0.9% (3)
No response	0.6% (2)
Experienced delays in multiple domains (i.e., lab work, imaging and medications)	
No delays/No requests	67.2% (234)
One domain	13.2% (46)
Two domains	12.9% (45)
All three domains	1.7% (6)
No response	4.9% (17)

### Quality of life scores and comparison with reference population data

Tables 3 and 4 summarize the quality of life scores (FCV-19S, EQ-5D-3L, PROMIS-29). Participants had a median FCV-19S score of 18.0 (IQR: 14.0–22.0) (Table 3). The participants median EQ-5D-3L score was 0.78 (IQR: 0.71–0.84), and median VAS was 71.0 (IQR: 50.0–80.0) (Table 3). Compared to a pre-pandemic PBC sample with a median EQ-5D-3L score of 0.89 (IQR: 0.83–0.92) and a median VAS score of 75 (IQR: 60–90), our PBC cohort had lower scores in both measures (S1 Table).

**Table 3:** Quality of life measures between participant sub-groups.

Quality of Life Measure	Entire PBC cohort (n = 348)	Experienced delay in any domain^a^ (n = 121)	Did not experience any delays (n = 225)		Mainly virtual/phone appointments^b^ (n = 264)	Mainly in-person appointments (n = 40)	
	Median (IQR^c^)	Median (IQR)	Median (IQR)	p-value	Median (IQR)	Median (IQR)	p-value
FCV-19S^d^	18.0 (14.0-22.0)	19.0 (15.0-24.0)	17.0 (13.0-22.0)	0.014	19.0 (14.0-22.0)	15.5 (11.5-23.0)	0.199
EQ-5D-3L^e^	0.78 (0.71-0.84)	0.77 (0.68-0.83)	0.78 (0.71-1.00)	0.009	0.78 (0.71-0.84)	0.78 (0.71-0.92)	0.542
EQ-5D VAS^f^	71.0 (50.0-80.0)	70.5 (50.0-80.0)	71.0 (50.0-80.0)	0.300	71.0 (50.0-80.0)	75.0 (53.0-81.0)	0.372
PROMIS^g^ Physical function	46.2 (39.0-56.9)	45.0 (38.7-56.8)	47.6 (40.1-56.9)	0.070	46.2 (39.7-56.9)	47.8 (40.1-56.9)	0.556
PROMIS Fatigue	57.2 (48.6-64.7)	60.7 (53.2-64.7)	55.2 (48.6-63.6)	0.001	57.2 (48.8-64.7)	57.9 (48.6-64.7)	0.836
PROMIS Anxiety	56.0 (47.9-61.4)	57.5 (51.3-63.2)	55.6 (40.3-59.6)	0.010	55.9 (47.9-61.4)	57.5 (40.3-61.7)	0.848
PROMIS Depression	52.2 (41.0-59.3)	53.0 (41.0-61.3)	52.1 (41.0-58.8)	0.207	52.5 (41.0-58.9)	54.6 (41.0-58.9)	0.882
PROMIS Sleep disturbance^h^	54.4 (49.4-60.4)	55.5 (51.1-61.9)	53.6 (48.0-59.3)	0.021	54.5 (49.6-60.2)	52.9 (46.3-59.2)	0.158
PROMIS Ability to participate in social roles and activities	46.0 (40.5-52.0)	44.2 (40.2-50.2)	47.1 (42.3-55.5)	0.008	46.0 (41.5-51.8)	47.2 (40.5-57.6)	0.357
PROMIS Pain interference	53.9 (41.6-61.3)	55.7 (41.6-61.3)	53.9 (41.6-61.3)	0.286	53.9 (41.6-61.3)	53.9 (41.6-61.3)	0.905
PROMIS Pain intensity scale	3.0 (0.0-5.0)	3.0 (1.0-5.0)	3.0 (0.0-5.0)	0.554	3.0 (0.0-5.0)	2.0 (0.0-6.0)	0.502

^a^Any of the three domains (lab, imaging, medications).

^b^Appointment type during the pandemic.

^c^Interquartile range.

^d^Fear of COVID-19 Scale.

^e^EuroQol 5-dimension 3-level.

^f^EuroQol 5-dimension visual analog scale.

^g^Patient-Reported Outcomes Measurement Instrument Survey.

^h^PROMIS Sleep disturbance was normally distributed and a two-sample t-test was used. Wilcoxon rank-rum test was used for all other variables.

**Table 4:** PBC PROMIS-29 scores compared to the United States reference population.

PROMIS Domain^a^	Median (IQR^b^)	p-value
Physical function	46.2 (39.0-56.9)	<0.001
Fatigue	57.2 (48.6-64.7)	<0.001
Anxiety	56.0 (47.9-61.4)	<0.001
Depression	52.2 (41.0-59.3)	0.004
Sleep disturbance	54.4 (49.4-60.4)	<0.001
Ability to participate in social roles and activities	46.0 (40.5-52.0)	<0.001
Pain interference	53.9 (41.6-61.3)	<0.001

^a^Reference population from the 2000 United States General Census (median T-score of 50).

^b^Interquartile range.

From the PROMIS-29, the participants had a median physical function score of 46.2 (IQR: 39.0–56.9), fatigue score of 57.2 (IQR: 48.6–64.7), anxiety score of 56.0 (IQR: 47.9–61.4), depression score of 52.2 (IQR: 41.0–59.3), sleep disturbance score of 54.4 (IQR: 49.4–60.4), ability to participate in social roles and activities score of 46.0 (IQR: 40.5–52.0), and pain interference score of 53.9 (IQR: 41.6–61.3) (Table 3). For the PROMIS numeric pain intensity scale the median response was 3 out of 10 (IQR: 0.0–5.0) (Table 3). Participants showed significant impairment across all domains compared to the USA general reference population (*p* < 0.01) (Table 4).

### Comparisons between patient sub-groups (i.e., delay experiences and appointment type)

Participants who experienced care delays (34.5%, n = 121) had significantly higher fear of COVID-19 (*p* = 0.014) and worse EQ-5D-3L quality of life scores (*p* = 0.009), compared to those without delays (Table 3). No differences in EQ-5D VAS scores were seen between these groups (*p* = 0.300). Participants with care delays had significantly worse fatigue (*p* = 0.001), anxiety (*p* = 0.010), sleep disturbances (*p* = 0.021) and a lower ability to participate in social roles and activities (*p* = 0.008), compared to those with no care delays (Table 3). There were no significant differences in quality of life between those with a majority of virtual visits versus those with in-person appointments (*p* > 0.05) (Table 3).

Patients who experienced care delays saw their hepatologist more frequently pre-pandemic than those without delays (*p* = 0.016) (S2 Table). Patients with care delays were more likely to have hepatology appointments at a tertiary centre (70.3%, n = 85) compared to a community clinic (23.1%, n = 28) prior to the pandemic, whereas non-delayed patients were split between tertiary centres (54.7%, n = 123) and community clinics (36.9%, n = 83) (*p* = 0.005). Patients with care delays were less likely to prefer future virtual appointments (16.5%, n = 20), compared to non-delayed patients (25.8%, n = 58) (*p* < 0.001). Most patients with virtual appointments during the pandemic were seen in tertiary centers (61.7%, n = 163) prior to the pandemic, while those with in-person appointments were more evenly split between tertiary centers (45.0%, n = 18) and community clinics (47.5%, n = 19) (*p* = 0.034).

### Predictors of future virtual appointments post pandemic

Univariable regression analyses revealed that patients with any care delay were less likely to prefer virtual care in the future (OR=0.43, 95%CI: 0.24–0.78, *p* = 0.005) (S3 Table). This remained significant in the multivariable analysis after adjusting for age (aOR=0.45, 95%CI: 0.24–0.84, *p* = 0.012). No other variables were significantly associated with preference for virtual care (*p* > 0.05).

### Qualitative analysis

#### Preference for type of medical appointment.

The first central theme from the patient focus groups was the importance of having choices in the delivery of medical appointments (i.e., virtual/in-person). One participant shared their preference for virtual appointments: “I loved being able to see my family doctor via Zoom. I don’t like the phone appointments because you don’t have that connection. But I think the video appointments were awesome because I could do it in my pjs and not leave the house. I think it fit into my schedule better, I could actually do my Zoom meeting with my doctor at work and just take a break then and do it” [Patient #5].

However, some participants felt that essential elements of healthcare, such as in-person communication, body language, and reassurance, were lost in virtual appointments. One participant emphasized the value of seeing their doctor in person: “I knew walking into her office she had a big smile on her face. So, when you see your specialist with a big smile on your face, I already knew I was in good shape” [Patient #2]. Another participant strongly expressed the need for in-person visits, that they “without a doubt” wanted “to see my specialist in person” [Patient #2]. In terms of the pandemic, they were willing to take any necessary precautions, stating: “You tell me what I have to do, you want to triple mask, I’ll put the triple mask [on], but I want to see you” [Patient #2]. Evidently, no single appointment format was universally preferred. Each participant had a preference based on their unique experiences, health status, geographical location, and other factors.

Hepatologists, however, made it clear that the shift to exclusively virtual care was not feasible. They stressed the difficulty of effectively monitoring patients in a virtual environment, citing their ethical and moral responsibilities to ensure patient safety,

### PBC care management

The second central theme focused on managing PBC and the intersection of three critical aspects of care: physician monitoring, proactive patient engagement, and collaborative efforts to drive research. Hepatologists were tasked with monitoring their patients’ health during a time of heightened stress on the healthcare system.

One hepatologist voiced concerns about lab work availability during the pandemic: “The other issue was going to get lab work, there was a lot of concern. First of all the labs were closed at the beginning for everything except for urgent things […] And so they couldn’t get in to get appointments. And so [it was] very hard to follow patients as we would normally follow them. I think it was a lot of concern that things might be missed” [Stakeholder #1]. A greater concern in the virtual setting was the lack of personal connection when monitoring patients. One hepatologist described: “I found it impossible to follow their blood results because it was random names. Who is this I’d say to myself, is this my patient?” [Stakeholder #2].

Conversely, many patients, particularly those who were engaged in their care, found benefits in accessing their medical information online. One participant explained their positive experience with telehealth platforms: “You can register an account and be able to see on your own your results. You can even download them as a PDF file […] or a spreadsheet if you’re savvy” [Patient #9]. This reflects the desire of patients to be more informed about their health without necessarily needing an in-person appointment. Another participant echoed this sentiment: “I love being able to see my labs and the trends, before I see my doctor. Gives you time to think and feel more comfortable and ask intelligent questions of [my] doctor” [Patient #10].

## Discussion

The pandemic restrictions and fear of COVID-19 infection drastically shifted healthcare delivery, forcing many patients and providers to primarily conduct virtual appointments. Our study aimed to understand the quality of life of PBC patients during the pandemic, in addition to describing their demographic, clinical and hepatologist appointment characteristics. Furthermore, we sought the perspectives of PBC patients and hepatologists on receiving and providing care during the pandemic.

Our findings suggest that PBC patients experienced elevated fear of COVID-19. Compared to a general reference population from New Zealand in the highest level of lockdown, our PBC cohort experienced higher fear of COVID-19 (FCV-19S mean = 15.6 and 18.1, respectively) [25]. Additionally, FCV-19S has been used in various settings where findings suggest that women and older adults with high anxiety levels are more likely to have higher fear of contracting COVID-19, which is consistent with our findings provided our sample demographics [26]. PBC patients are at higher risk for severe COVID-19 infection, with studies indicating they are over two times more susceptible to COVID-19-related hospitalizations [27]. This increased susceptibility to severe COVID-19 cases and hospitalizations may have added stress and strain for both the patients and hepatologists, further complicating the delivery of safe care during the pandemic.

Janssen et al. (2018) reported pre-pandemic EQ-5D-3L mean scores of 0.83 in PBC patients, which indicates better quality of life compared to our cohort mean of 0.78 and emphasizes the negative impact of COVID-19 on PBC patients’ quality of life [28]. Further, our cohort mean EQ-5D VAS participant score was 65.0, which is lower than the pre-pandemic healthy reference population mean scores of 79.0 from Poland and 70.0 from Italy [28]. Additionally, Cortesi et al. (2020) completed a cross-sectional quality of life study among patients with chronic liver diseases from March 2011 to November 2012, where PBC patients scored a median EQ-5D-3L score of 0.89 and EQ VAS score of 75 [22]. When compared to our participants EQ-5D-3L median of 0.78 and EQ VAS of 71, we again see the potential impact of COVID-19 on reducing PBC patients’ quality of life. These findings suggest that during the pandemic Canadian PBC patients experience an overall reduced quality of life when compared to healthy and pre-pandemic PBC populations.

Our participants scored significantly worse across all PROMIS domains in comparison to the reference population from the 2000 USA general census, particularly in the fatigue and anxiety domains [19]. These results corroborate previous studies linking PBC with chronic symptoms of fatigue and anxiety, and contribute to social, emotional, and cognitive impairments [29–32].

We compared quality of life measures between those who experience any care delays and those who experienced no care delays. Participants who experienced any care delays had higher fear of COVID-19 and worse quality of life scores with respect to their overall EQ-5D-3L score, fatigue, anxiety, sleep disturbances, and ability to participate in social roles and activities. This highlights an important factor with regards to an individual’s care, in that experiencing delays in care through accessibility issues, geographical barriers, or self-inflicted delays due to anxiety around contracting COVID-19, can impact a patient’s overall quality of life. This finding aligns with other disease populations as reported by Shah et al. (2023), who found that breast cancer patients who experienced care delays during the pandemic reported moderate to extreme anxiety [33]. These results underline the importance of timely care, as care delays may have a detrimental impact on short- and long-term health outcomes.

Regarding appointment type during the pandemic, we compared quality of life measures of patients who had the majority of their appointments virtually with those patients who had the majority of their appointments in-person. This comparison did not show any significant differences in quality of life. These results could be explained, at least in part, by the observation that healthcare providers commonly determine what type of appointment is necessary for the patient, depending on their health status.

The pandemic resulted in a striking shift in healthcare delivery, with most PBC-related care moving to a virtual platform. This shift mirrors trends observed in other chronic diseases, such as liver cirrhosis, and aligns with a broader national trend of increased virtual healthcare utilization during the pandemic [12,34]. Glazier et al. (2021) found that primary care physician office visits in Ontario, Canada, declined by 79.1% between March to July 2020, in comparison to rates from 2019, and that virtual visits comprised of 71.1% of all primary care visits during that time [12]. Our findings suggest that, although virtual care was predominant during the pandemic and provided flexibility and convenience for many patients, a large portion still preferred in-person consultations for better communication and emotional reassurance. Further to this, our study sought to identify factors that were associated with a preference for future virtual care. Patients who experienced any care delays during the pandemic were less likely to prefer virtual care moving forward. This may reflect that the emotional and logistical challenges encountered during virtual care visits, particularly in cases of experiencing other care delays, may shape patients’ preferences for future healthcare delivery.

Our study highlights the importance of offering patients a choice between in-person and virtual appointments, tailored to their health needs and personal circumstances. This personalized approach should be balanced with the hepatologist’s ethical responsibility to offer appropriate and timely care, depending on disease stability and patient engagement. It is essential to understand the role of virtual healthcare and other online platforms as being a complement to the development of improved patient-centric care.

Our study has some limitations. The response rate to the quantitative survey (<50%) limits the generalizability to the whole PBC patient population across Canada. The Canadian PBC Society contact list (i.e., those with a valid email or mailing address) consisted mainly of individuals from British Columbia (22.0%), Alberta (15.2%) and Ontario (36.1%), which may skew our results to better reflect the experiences and preferences from individuals in those provinces. Although we contacted patients using two different methods (i.e., email and postage mail), we may have missed a subgroup of patients that do not use either method of communication, or who may not have time to fill out the survey due to competing priorities. The FCV-19S was administered after COVID-19 vaccinations became available across Canada, which may have had an impact on the level of fear experienced by participants. The EQ-5D-3L and PROMIS-29 survey data were compared with reference population data that was collected pre-pandemic. Recall bias may be introduced due to the timeline of our data collection events. However, the nature of our study focused on general clinical care and quality of life experiences during the pandemic, rather than seeking specific information about events. Although the study design attempts to account for key factors relating to clinical care and quality of life experiences during the pandemic, residual confounders (i.e., underlying medical conditions) cannot be excluded entirely and may influence our findings. Given the cross-sectional design of our study, temporality cannot be established and causal inferences cannot be made.

Despite these limitations, our study had many strengths that add to the validity and reliability of our results. This study was a mixed-methods design, which provided both quantitative data and qualitative insights into patient and stakeholder perspectives. Our sampling frame included PBC patients from across Canada, which may widen the geographical scope of our findings. Our qualitative data included the perspective of hepatologists from two different provinces, which was used to build upon the patients’ viewpoint to ensure their preferences and expectations were feasible and realistic from a provider’s point of view. The quality of life surveys in this study were specifically chosen to reflect different facets of life quality and to allow for comparison to other populations. Our study was novel in that the PROMIS-29 and FCV-19S questionnaires have not been used in the PBC population to date, thus, providing baseline results for future studies with the caveat that our survey was administered during the pandemic.

In conclusion, our study underscores the diverse experiences of PBC patients during the pandemic and suggests that no single mode of care delivery would suit all patients post-pandemic. The impact of the pandemic on patient quality of life was evident, with many patients reporting a marked decline in their physical and emotional health. Moving forward, offering patients a choice between virtual and in-person care, while accounting for their health status, engagement in care, and emotional well-being, will be essential in improving outcomes and enhancing patient satisfaction.

## Supporting information

S1. File PBC Care Delivery Questionnaire. (DOCX)

S1 Table Euro-Qol 5D-3L and Visual Analog Scale (EQ-5D VAS) sample scores compared to pre-pandemic PBC reference sample. a. Wilcoxon sign-rank test**. b.** Visual analog scale**. c.** Interquartile range. d. Standard deviation.
(DOCX)

S2 Table Comparing demographic and clinical characteristics between any delay versus no delay & virtual versus in-person appointments during the pandemic. a. Any of the three domains (lab, imaging, medications). b. Appointment type during the pandemic. c. Interquartile range. d. Decompensated liver conditions included ascites, variceal bleeding, jaundice and hepatic encephalopathy.
(DOCX)

S3 Table Characteristics associated with preference for virtual care in the future (n = 247). **a.** Odds ratio. b. Confidence interval. c. Decompensated liver conditions included ascites, variceal bleeding, jaundice and hepatic encephalopathy. d. Fear of COVID-19 Scale. e. EuroQol 5-dimension 3-levelVisual analog scale. f. Patient-Reported Outcomes Measurement Instrument Sur.vey.
(DOCX)
