# Supplementary material for: Evaluating the clinical care, quality of life and overall experiences of patients with primary biliary cholangitis (PBC) during the pandemic: A Canadian mixed-methods study
Source: PLoS One. 2026 Jan 9;21(1):e0340475. doi: 10.1371/journal.pone.0340475 (PMC12788631; doi:10.1371/journal.pone.0340475)

**Supplementary Materials:** Evaluating the clinical care, quality of life and overall experiences of patients with primary biliary cholangitis (PBC) during the pandemic: A Canadian mixed-methods study.

Elizabeth Baguley^1,2¶^ | Madelyn Knaub^3&^ | Jessica VanDyke^3&^ | Gideon Hirschfield^4&^ | Mark G. Swain^1&^ | Gail Wright^5&^ | Deirdre McCaughey^2,3&^ | Abdel Aziz Shaheen^1,2¶*^

^1^ University of Calgary, Division of Gastroenterology and Hepatology, Calgary, Alberta, Canada

^2^ University of Calgary, Department of Community Health Sciences, Calgary, Alberta, Canada

^3^ University of Calgary, Ward of the 21^st^ Century (W21C), Calgary, Alberta, Canada

^4^ Toronto Centre for Liver Disease, Division of Gastroenterology and Hepatology, University of Toronto, Toronto, Ontario, Canada

^5^ Canadian PBC Society, Toronto, Ontario, Canada

***** Corresponding author

Email: [aashahee@ucalgary.ca](mailto:aashahee@ucalgary.ca) (AS)

^¶^ These authors contributed equally to this work.

^&^ These authors also contributed equally to this work.

**Supporting Information**

**S1. File. PBC Care Delivery Questionnaire**


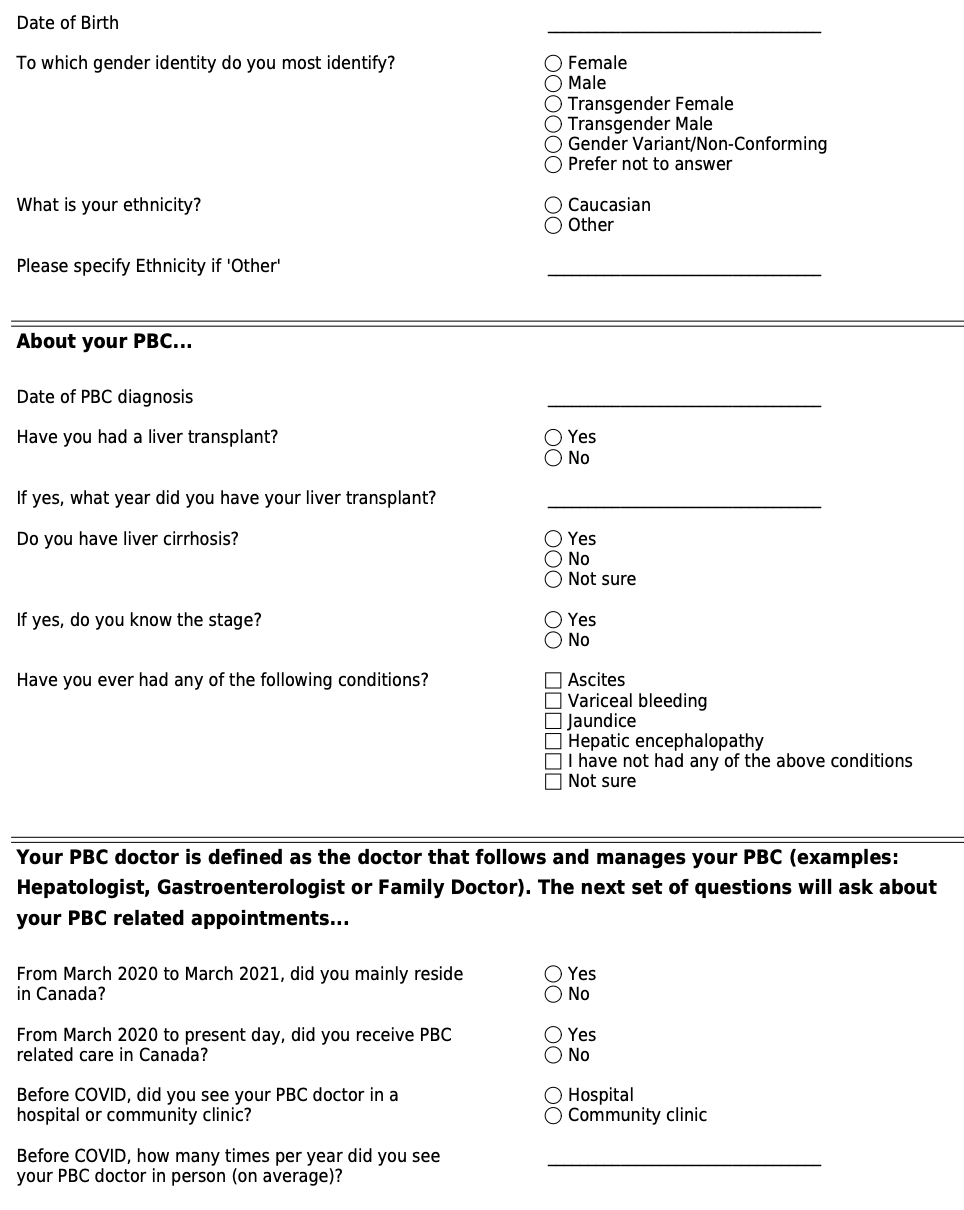


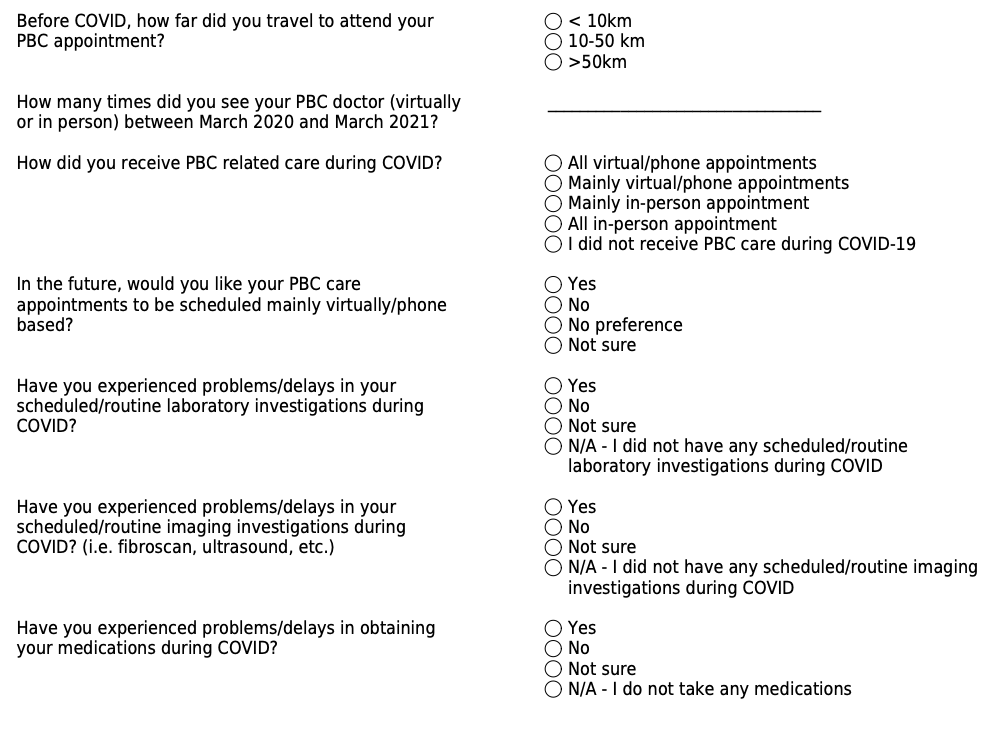

Supplement: S1. File — (DOCX) [file pone.0340475.s001.docx]
