# Supplementary material for: Evaluating the clinical care, quality of life and overall experiences of patients with primary biliary cholangitis (PBC) during the pandemic: A Canadian mixed-methods study
Source: PLoS One. 2026 Jan 9;21(1):e0340475. doi: 10.1371/journal.pone.0340475 (PMC12788631; doi:10.1371/journal.pone.0340475)
Supplement: S1 Table — a. Wilcoxon sign-rank test. b. Visual analog scale. c. Interquartile range. d. Standard deviation. (DOCX) [file pone.0340475.s002.docx]

**S1 Table. Euro-Qol 5D-3L and Visual Analog Scale (EQ-5D VAS) sample scores compared to pre-pandemic PBC reference sample**

|  | EQ-5D-3L | EQ-5D VAS^a^ |
| --- | --- | --- |
| Sample median score (IQR^b^) | 0.78 (0.71 – 0.84) | 71 (50 – 80) |
| Sample mean score (SD^c^) | 0.78 (0.16) | 65.00 (19.81) |
| Reference sample median (IQR) from pre-pandemic PBC population | 0.89 (0.83 – 0.92) | 75 (60 – 90) |

1. Visual analog scale
2. Interquartile range
3. Standard deviation
