# Supplementary material for: Evaluating the clinical care, quality of life and overall experiences of patients with primary biliary cholangitis (PBC) during the pandemic: A Canadian mixed-methods study
Source: PLoS One. 2026 Jan 9;21(1):e0340475. doi: 10.1371/journal.pone.0340475 (PMC12788631; doi:10.1371/journal.pone.0340475)
Supplement: S2 Table — a. Any of the three domains (lab, imaging, medications). b. Appointment type during the pandemic. c. Interquartile range. d. Decompensated liver conditions included ascites, variceal bleeding, jaundice and hepatic encephalopathy. (DOCX) [file pone.0340475.s003.docx]

**S2 Table. Comparing demographic and clinical characteristics between any delay versus no delay & virtual versus in-person appointments during the pandemic**

|  | **Experienced delay in any domain^a^ (n=121)** | **Did not experience any delays (n=225)** |  | **Mainly virtual/ phone appointments^b^ (n=264)** | **Mainly in-person appointments (n=40)** |  |
| --- | --- | --- | --- | --- | --- | --- |
| **Continuous characteristics** | **Median (IQR^c^)** | | **p-value** | **Median (IQR)** | | **p-value** |
| Age (years) | 61.0 (55.1-70.3) | 63.7 (57.4-73.0) | 0.078 | 62.8 (55.7-70.2) | 63.4 (57.4-74.5) | 0.739 |
| Time since PBC diagnosis (years) | 8.3 (4.9-17.7) | 9.6 (4.6-18.2) | 0.470 | 8.8 (4.6-17.0) | 9.4 (3.2-19.2) | 0.907 |
| Frequency that participant saw hepatologist before the pandemic | 2 (1-2) | 1 (1-2) | 0.016 | 2 (1-2) | 2 (1-2) | 0.748 |
| Frequency that participant saw hepatologist during the pandemic | 2 (1-2) | 1 (1-2) | 0.688 | 2 (1-2) | 2 (1-2.5) | 0.946 |
| **Categorical characteristics** | **% (n)** | |  | **% (n)** | |  |
| Gender  Female  Male  Gender Variant/Non-Conforming  No response | 94.2% (114)  5.0% (6)  0.8% (1)  0% (0) | 93.8% (211)  4.9% (11)  0% (0)  1.3% (3) | 0.398 | 95.1% (251)  3.8% (10)  0.4% (1)  0.8% (2) | 87.5% (35)  12.5% (5)  0% (0)  0% (0) | 0.059 |
| Ethnicity  Caucasian  Other  No response | 87.6% (106)  8.3% (10)  4.1% (5) | 88.5% (199)  7.1% (16)  4.4% (10) | 0.704 | 89.4% (236)  6.8% (18)  3.8% (10) | 82.5% (33)  10.0% (4)  7.5% (3) | 0.423 |
| Received liver transplant during study period  Yes  No  No response | 2.5% (3)  96.7% (117)  0.8% (1) | 0.9% (2)  98.7% (222)  0.4% (1) | 0.235 | 1.5% (4)  98.1% (259)  0.4% (1) | 2.5% (1)  95.0% (38)  2.5% (1) | 0.634 |
| Presence of liver cirrhosis  Yes  No  Not sure  No response | 24.8% (30)  52.9% (64)  19.8% (24)  2.5% (3) | 25.8% (58)  55.1% (124)  18.2% (41)  0.9% (2) | 0.909 | 27.3% (72)  53.4% (141)  17.8% (47)  1.5% (4) | 22.5% (9)  60.0% (24)  15.0% (6)  2.5% (1) | 0.693 |
| Diagnosis of decompensated liver cirrhosis conditions^d^  Never  1 decompensated liver condition  >1 decompensated liver condition  Unsure  No response | 63.6% (77)  11.6% (14)  9.1% (11)  13.2% (16)  2.5% (3) | 73.8% (166)  7.6% (17)  4.0% (9)  9.3% (21)  5.3% (12) | 0.069 | 74.2% (196)  7.2% (19)  5.7% (15)  8.3% (22)  4.6% (12) | 60.0% (24)  15.0% (6)  10.0% (4)  12.5% (5)  2.5% (1) | 0.160 |
| Hepatologist location  Tertiary centre affiliated clinic (i.e., Hospital)  Community clinic  No response | 70.3% (85)  23.1% (28)  6.6% (8) | 54.7% (123)  36.9% (83)  8.4% (19) | 0.005 | 61.7% (163)  31.1% (82)  7.2% (19) | 45.0% (18)  47.5% (19)  7.5% (3) | 0.034 |
| Hepatologist appointment type during the pandemic  Majority (>50%) virtual/phone  Majority (>50%) in-person  Did not receive care  No response | 81.0% (98)  8.3% (10)  9.9% (12)  0.8% (1) | 73.8% (166)  13.3% (30)  12.4% (28)  0.4% (1) | 0.256 | -- | -- |  |
| Preference for future virtual/phone appointments  Yes  No  No preference  Not sure  No response | 16.5% (20)  62.0% (75)  9.9% (12)  11.6% (14)  0% (0) | 25.8% (58)  41.8% (94)  24.0% (54)  8.4% (19)  0% (0) | <0.001 | 24.6% (65)  47.4% (125)  19.3% (51)  8.7% (23)  0% (0) | 15.0% (6)  57.5% (23)  25.0% (10)  2.5% (1)  0% (0) | 0.224 |
| Experienced delays in routine lab work  Yes  No  Not sure  No lab work requested  No response | -- | -- |  | 22.4% (59)  73.9% (195)  0.8% (2)  2.7% (7)  0.4% (1) | 20.0% (8)  77.5% (31)  0% (0)  2.5% (1)  0% (0) | 0.929 |
| Experienced delays in imaging investigations  Yes  No  Not sure  No imaging requested  No response | -- | -- |  | 25.8% (68)  53.8% (142)  2.3% (6)  18.2% (48)  0% (0) | 17.5% (7)  67.5% (27)  2.5% (1)  12.5% (5)  0% (0) | 0.426 |
| Experienced delays in obtaining medications  Yes  No  Not sure  No response | -- | -- |  | 6.8% (18)  92.8% (245)  0.4% (1)  0% (0) | 0% (0)  95.0% (38)  5% (2)  0% (0) | 0.006 |
| Experienced delays in multiple domains (i.e., lab work, imaging and medications)  No delay/No request  One domain  Two domains  All three domains  No response | -- | -- |  | 65.9% (174)  15.5% (41)  12.9% (34)  2.3% (6)  3.4% (9) | 72.5% (29)  7.5% (3)  12.5% (5)  0% (0)  7.5% (3) | 0.438 |

1. Any of the three domains (lab, imaging, medications)
2. Appointment type during the pandemic
3. Interquartile range
4. Decompensated liver conditions included ascites, variceal bleeding, jaundice and hepatic encephalopathy
