# Supplementary material for: Evaluating the clinical care, quality of life and overall experiences of patients with primary biliary cholangitis (PBC) during the pandemic: A Canadian mixed-methods study
Source: PLoS One. 2026 Jan 9;21(1):e0340475. doi: 10.1371/journal.pone.0340475 (PMC12788631; doi:10.1371/journal.pone.0340475)
Supplement: S3 Table — a. Odds ratio. b. Confidence interval. c. Decompensated liver conditions included ascites, variceal bleeding, jaundice and hepatic encephalopathy. d. Fear of COVID-19 Scale. e. EuroQol 5-dimension 3-levelVisual analog scale. f. Patient-Reported Outcomes Measurement Instrument Sur.vey. (DOCX) [file pone.0340475.s004.docx]

**S3 Table. Characteristics associated with preference for virtual care in the future (n=247)**

| **Characteristics** | **Univariable Logistic Regression (OR^a^ [95%CI^b^])** | **Multivariable Logistic Regression (OR [95%CI])** |
| --- | --- | --- |
| Age | 0.98 (0.95-1.00) | 0.97 (0.95-1.00) |
| Length of time with PBC | 1.00 (0.97-1.02) | -- |
| Diagnosis of cirrhosis  No  Yes  Not sure | Reference  0.61 (0.31-1.21)  1.10 (0.55-2.19) | -- |
| Decompensated liver conditions^c^  Never  One condition  Two or more conditions  Not sure | Reference  1.08 (0.45-2.61)  0.53 (0.14-1.96)  0.67 (0.28-1.60) | -- |
| Times seen by hepatologist in the pandemic  Zero  Once  Two or more | Reference  1.11 (0.45-2.72)  1.96 (0.85-4.49) | -- |
| Experienced any delay with PBC care | 0.43 (0.24-0.78) | 0.45 (0.24-0.84) |
| PBC care in community clinic | 1.01 (0.56-1.84) | -- |
| Distance to hepatologist  <10km  10-50km  >50km | Reference  0.89 (0.47-1.67)  0.89 (0.43-1.83) | -- |
| Appointment type during the pandemic  Mainly virtual visits  Mainly in-person visits  Did not receive care | Reference  0.50 (0.19-1.29)  0.58 (0.22-1.51) | -- |
| FCV-19S^d^ | 1.01 (0.96-1.06) | -- |
| EQ-5D-3L^e^ | 1.80 (0.32-10.01) | -- |
| EQ-5D VAS^f^ | 1.01 (0.99-1.02) |  |
| PROMIS^g^ Physical Function | 1.01 (0.98-1.04) | -- |
| PROMIS Anxiety | 0.98 (0.95-1.00) | -- |
| PROMIS Depression | 0.98 (0.96-1.01) | -- |
| PROMIS Fatigue | 1.00 (0.97-1.03) | -- |
| PROMIS Sleep Disturbance | 0.99 (0.96-1.02) | -- |
| PROMIS Ability to Participate in Social Roles and Activities | 1.01 (0.98-1.04) | -- |
| PROMIS Pain Interference | 1.01 (0.98-1.04) | -- |
| PROMIS Pain Intensity Scale | - 1. (0.92-1.14) | -- |

# Odds ratio

1. Confidence interval
2. Decompensated liver conditions included ascites, variceal bleeding, jaundice and hepatic encephalopathy
3. Fear of COVID-19 Scale
4. EuroQol 5-dimension 3-level
5. Visual analog scale
6. Patient-Reported Outcomes Measurement Instrument Survey
